# *Caenorhabditis elegans* DAF-2 as a Model for Human Insulin Receptoropathies

**DOI:** 10.1534/g3.116.037184

**Published:** 2016-11-15

**Authors:** David A. Bulger, Tetsunari Fukushige, Sijung Yun, Robert K. Semple, John A. Hanover, Michael W. Krause

**Affiliations:** * Laboratory of Molecular Biology, National Institute of Diabetes and Digestive and Kidney Diseases, National Institutes of Health, Bethesda, Maryland 20878; † Institute of Metabolic Science, University of Cambridge, CB2 0QQ, UK; ‡ Laboratory of Molecular and Cellular Biology, National Institute of Diabetes and Digestive and Kidney Diseases, National Institutes of Health, Bethesda, Maryland 20878

**Keywords:** DAF-2, INSR, CRISPR, Million Mutation Project, dauer

## Abstract

Human exome sequencing has dramatically increased the rate of identification of disease-associated polymorphisms. However, examining the functional consequences of those variants has created an analytic bottleneck. Insulin-like signaling in *Caenorhabditis elegans* has long provided a model to assess consequences of human insulin signaling mutations, but this has not been evaluated in the context of current genetic tools. We have exploited strains derived from the Million Mutation Project (MMP) and gene editing to explore further the evolutionary relationships and conservation between the human and *C. elegans* insulin receptors. Of 40 MMP alleles analyzed in the *C. elegans* insulin-like receptor gene DAF-2, 35 exhibited insulin-like signaling indistinguishable from wild-type animals, indicating tolerated mutations. Five MMP alleles proved to be novel dauer-enhancing mutations, including one new allele in the previously uncharacterized C-terminus of DAF-2. CRISPR-Cas9 genome editing was used to confirm the phenotypic consequence of six of these DAF-2 mutations and to replicate an allelic series of known human disease mutations in a highly conserved tyrosine kinase active site residue, demonstrating the utility of *C. elegans* for directly modeling human disease. Our results illustrate the challenges associated with prediction of the phenotypic consequences of amino acid substitutions, the value of assaying mutant isoform function *in vivo*, and how recently developed tools and resources afford the opportunity to expand our understanding even of highly conserved regulatory modules such as insulin signaling. This approach may prove generally useful for modeling phenotypic consequences of candidate human pathogenic mutations in conserved signaling and developmental pathways.

The evolutionarily conserved insulin/IGF-1 signaling (IIS) pathway is a critical regulator of growth, development, and longevity (Taniguchi *et al.* 2006). In humans, excess insulin signaling is associated with overgrowth and cancer, while loss of signaling results in insulin resistance and diabetes (Lindhurst *et al.* 2012). The recent expansion of human polymorphisms identified in genes encoding insulin signaling molecules is generating a potentially valuable resource for the diagnosis of metabolic disease. Full understanding of the functional consequences of these polymorphisms ideally requires models providing *in vivo* context, that is, in which the complete developmental and metabolic program is an integral part of the phenotype.

The nematode *Caenorhabditis elegans* has proven to be an excellent animal model in which to examine insulin-like signaling. In *C. elegans*, reduction or partial loss of IIS has pleiotropic effects, including altered stress responses and increased longevity (Gottlieb and Ruvkun 1994). The worm IIS also controls entry into the dauer stage under adverse conditions (*e.g.*, overcrowding, elevated temperature, and starvation), providing a convenient readout for the strength of IIS signaling. Genetic screens for dauer formation defective (Daf-d), and dauer formation constitutive (Daf-c), mutants have identified alleles in *C. elegans* genes encoding each of the IIS pathway components that have helped to define and understand this important signaling cascade (Gottlieb and Ruvkun 1994; Malone and Thomas 1994; Hu 2007).

Forward genetic screens in *C. elegans* for Daf-d or Daf-c mutants, and their suppressors, impose an inherent bias due to the selection for a specific phenotype. It is unclear to what extent that bias has limited genetic analysis of the IIS pathway. For example, the strong phenotypes selected by such forward screens may miss those alleles that more closely mimic human disease-associated polymorphisms that result in more subtle metabolic phenotypes. One approach that minimizes the possible bias of phenotypic selection is to isolate random mutations in pathway components prior to assaying the mutant alleles for phenotypes. This approach also allows for the identification of silent mutations that provide information on tolerated alleles in pathway components. The Million Mutation Project (MMP) in *C. elegans* provides such a resource, consisting of a distributed collection of genetic alleles generated by chemical mutagenesis and selected only for viability at room temperature (Thompson *et al.* 2013). Each MMP strain has been clonally selected and expanded for ten generations followed by whole genome sequencing, providing a rich array of nonlethal homozygous variants within most genes of interest that are available for further study.

We have taken advantage of the MMP resource to further investigate IIS, focusing on novel alleles of the single insulin-like receptor in the worm, DAF-2. The DAF-2 protein has already been extensively analyzed using the mutants obtained through genetic screens, which resulted in a classification of the *daf-2* mutants into Class I and pleiotropic Class II based on the constellation of phenotypes (Gems *et al.* 1998; Patel *et al.* 2008). Genetic screens have isolated several temperature-sensitive, reduction-of-function alleles in *daf-2*, demonstrating that reduced IIS activity results in animals entering the dauer stage under conditions in which wild type animals would not. This well-defined, graded response makes DAF-2 an ideal candidate to test the value of the MMP resource in providing additional insight into gene function. We have taken advantage of a very sensitive high temperature-induced dauer assay (Ailion and Thomas 2003) to screen MMP alleles distributed throughout the daf-2 coding region for dauer-related phenotypes. Of the 40 *daf-2* MMP alleles we analyzed, 35 retained IIS function and were indistinguishable from wild-type animals, providing the first list of tolerated substitutions. Importantly, five MMP alleles defined novel dauer-enhancing mutations. Using CRISPR-Cas9 genome editing, we have confirmed the phenotype for many of these alleles, and replicated an allelic series of known human disease mutations in a highly conserved tyrosine kinase active site residue. Our results capitalize on existing MMP alleles to expand our understanding of residues critical for DAF-2 function, while demonstrating the usefulness of *C. elegans* genomics to model human alleles for functional studies in the context of a developing and metabolically active animal system.

## Materials and Methods

### Nematode culture and strains

#### Strain construction:

Standard *C. elegans* maintenance procedures were followed (Brenner 1974). N2 (Bristol) wild-type and RW3625 *let-805*(*st456*)/*qC1* [*dpy-19*(*e1259ts*) *glp-1*(*q339*)] were used as negative controls and for backcrossing. The following strains were obtained using microinjection of the pDD162 plasmid-based CRISPR-Cas9 system (Dickinson *et al.* 2013; Paix *et al.* 2014) with a coedited *dpy-10(cn64)* marker (Arribere *et al.* 2014) (Supplemental Material, Table S4): *daf-2(gv51[P155L])*, *daf-2(gv52[S341P])*, *daf-2(gv53[A746T*; *del])*, *daf-2(gv54[A1015V])*, *daf-2(gv55[A1391T])*, *daf-2(gv56[A1391V])*, *daf-2(gv57[A1391E])*, and *daf-2(gv58[A1729V])*.

### Sequencing of mutant alleles

Note that all INSR and DAF-2 amino acid designations refer to the preproreceptor sequence. All *daf-2* exons were sequenced using nested PCR followed by Sanger sequencing with 13 amplicons used to cover the entire coding region (Table S1). For strains with previously identified mutations, only the relevant exon was sequenced; MMP allele sequences were available from both the WormBase (http://www.wormbase.org/) and MMP (http://genome.sfu.ca/mmp/) websites. Strains were continuously verified after genetic crosses and propagation using Sanger sequencing of the affected exon region.

Two *daf-2* mutants, *sa753* and *sa875*, isolated previously in a high-temperature dauer positive selection screen (Ailion and Thomas 2003), were found by whole gene sequencing of *daf-2* to have the same DAF-2(P470L) substitution, which has been previously reported as one of the two mutations found in *mg43*, DAF-2(C401Y; P470L) (Kimura *et al.* 1997).

### High-temperature dauer assay

Gravid adults (1–10) were grown at 20° and allowed to lay embryos overnight (12 hr), the adults were removed and the plates were shifted to either high temperature (27.2°) or a range of typical incubation temperatures (15, 20, and 25°) (Ailion and Thomas 2003). After 72 hr at the assay temperature, the progeny were scored by counting the number of dauers, early larval stage animals, and adults on each plate. Worms that had crawled up the edge of the plate were also counted (Lee *et al.* 2011). Some high temperature dauer plates were left at 27.2° for another 72 hr, and then recovered at 15° for 7 d. These plates were then scored for having sterile adults with dead progeny (wild-type), dauers that recovered as fertile adults, or live dauers that remained arrested.

### Molecular dynamics simulation

Molecular dynamics simulations were performed using NAMD version 2.9 on the human INSR tyrosine kinase (PDB: 4XLV) and its point mutation variants. The point mutations were induced using PyMol Molecular Graphics System, Version 1.7.6.0 Schrödinger, LLC. CHARMM Version 27 force field parameters were used to generate solvated psf files with a 15 Å additional layer of water molecules from the most outer portion of the protein (Brooks *et al.* 2009). The time step of the simulation was 1 fs. Periodic boundary condition was used. For equilibration, 5000 steps of energy minimization with conjugate gradient method has been performed initially, then the temperature was raised from absolute zero to 37° in 1° intervals using Langevin dynamics, with 1 ps of simulation at the temperature. Then, five cycles of 10 ps simulation with 2000 steps of energy minimization were performed. Each simulation was performed for an additional 20 ns (videos provided in File S2, File S3, File S4, and File S5).

### Data availability

Strains are available upon request. Table S1 contains primers used for *daf-2* sequencing. Table S2 contains a complete list of the dauer assay results by independent isolate. Table S3 contains dauer-associated mutations in prebackcrossed MMP strains. Table S4 contains the CRISPR-Cas9 gRNA and dsDNA repair template sequences with associated protocols. Table S5 contains SIFT, Polyphen2, AlignGVGD, and PROVEAN results. Table S6 contains quality scores and metrics for the DAF-2 I-TASSER model (Figure 4). Table S7 contains PDB IDs with supporting data for the human tyrosine kinase structural analysis. Figure S1 contains the human-to-worm sequence alignment annotated with the MMP mutations. File S1 contains the complete sequence alignment file. File S2, File S3, File S4, and File S5 contain molecular dynamics simulations highlighting the ATP movements in the tyrosine kinase binding pocket of normal and mutant human INSR. High resolution videos are available upon request. File S6 contains the human tyrosine kinase active site alignment. File S7, File S8, File S9, File S10, File S11, File S12, and File S13 contain the DAF-2 I-TASSER model PDB files (Figure 4).

## Results

**Figure 1:**
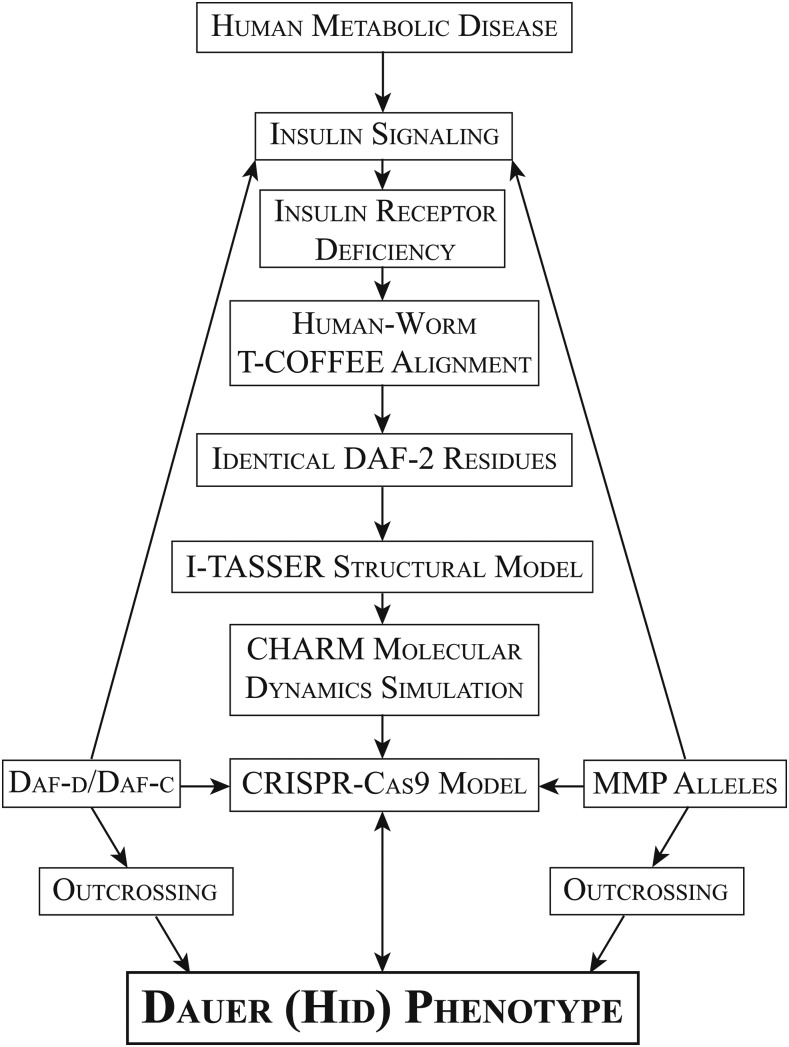
Strategy and analysis pipeline exploiting human insulin-receptoropathy-associated polymorphisms and linking them to emerging *C. elegans* genetic tools. Among the components of the insulin signaling pathway, the human insulin receptor was chosen for its high degree of sequence similarity with *C. elegans* DAF-2. A combination of existing dauer mutants (Daf-defective and Daf-constitutive), MMP alleles and CRISPR-Cas9 generated alleles were coupled with a recursive verification of a high-temperature induction of dauer (Hid) phenotype to provide a detailed look at conserved regions of the human insulin receptor and the functional consequences of conservative and nonconservative replacements of critical residues in the tyrosine kinase domain.

Recent advances in worm genetics and genomics have accelerated the ability to generate, map, and characterize mutant alleles. We devised a pipeline to examine the extent to which these technological advances could be applied to a well-characterized and evolutionarily conserved pathway to both gain a deeper understanding of structure-function relationships and to model human disease. The insulin-like signaling pathway was chosen for a detailed analysis. As shown in Figure 1, this pipeline was designed to use information derived from existing mutations in the human IIS as a starting point and to use *C. elegans* genetics to model mutations in conserved regions of components of this well-studied pathway. The strategy used bioinformatic tools coupled with allele selection and gene editing to examine a robust insulin-dependent phenotype in an intact organism, focusing on a high-throughput dauer assay.

### DAF-2 MMP alleles as a proof-of-principle target

Alleles available from the MMP were selected solely on the basis of survival at room temperature (Thompson *et al.* 2013). One goal of this study was to determine if MMP alleles would have value in further understanding the IIS pathway. In humans, defects in insulin signaling can present with a wide range of phenotypic consequences for the affected patient. Modeling this phenotypic spectrum may require the identification of more subtle phenotypes than traditional screens allow.

**Figure 2:**
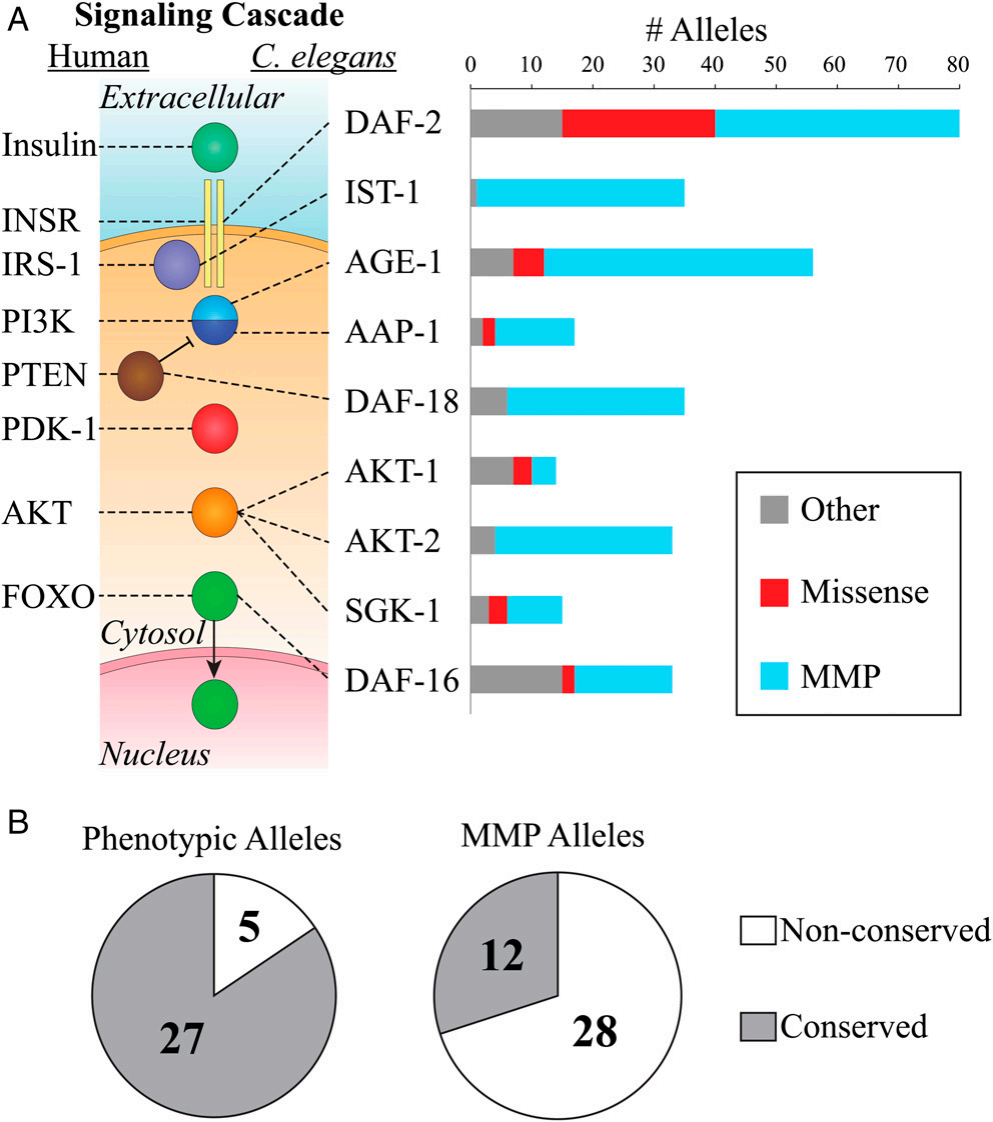
The evolutionarily conserved insulin-like signaling pathway members, associated alleles, and their distributions. (A) The *C. elegans* and human insulin signaling pathways are highly conserved from the DAF-2/INSR insulin-like receptor all the way to the DAF-16/FOXO nuclear transcription factor [pathway adapted from Christensen *et al.* (2002)]. Only the pathway proteins associated with human insulin resistance and/or *C. elegans* dauer phenotypes are shown in this simplified diagram. Dauer-associated alleles identified in previous genetic screens are indicted in red and gray bars labeled “Missense” and “Other,” respectively. MMP alleles are shown by blue bars. (B) Distinct distributions of phenotypically selected *vs.* MMP DAF-2 alleles among conserved and nonconserved residues. DAF-2 alleles identified by phenotypic selection appear to be biased toward substitutions in evolutionarily conserved residues, as defined in Table 1. In contrast, MMP alleles that were selected only for viability at room temperature do not seem to exhibit this bias.

To fully understand the scope of IIS pathway mutants available in *C. elegans*, we interrogated WormBase (v245) for all known alleles in evolutionarily conserved IIS pathway genes (Howe *et al.* 2016), including genetic mutants, deletion alleles, and nonsynonymous single nucleotide polymorphisms (nsSNPs) identified by the MMP. As Figure 2A demonstrates, the MMP nsSNPs greatly expand the range of mutations available for the components of the IIS pathway. We focused this study on the sole *C. elegans* insulin-like receptor, DAF-2, for several reasons: (1) 40 new missense alleles were present in the MMP collection, allowing us to further assess DAF-2 structure-function; (2) reduction of DAF-2 activity results in an easily scored dauer phenotype in a dose-dependent manner; (3) 25 mutants resulting in a dauer, or more severe larval arrest, phenotype with a known genotype have previously been characterized; and (4) the protein is closely related to the human insulin receptor (INSR), which has been extensively characterized in patients with severe insulin resistance, and morbidity due to INSR mutations (Gems *et al.* 1998; Patel *et al.* 2008).

**Figure 3:**
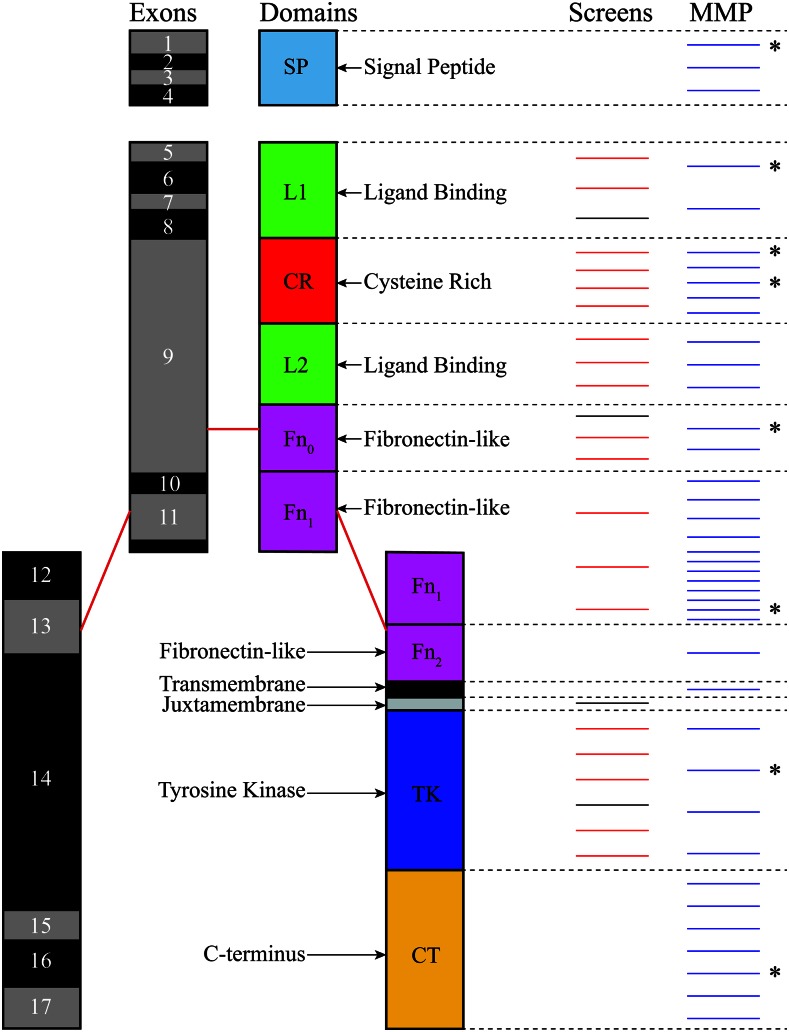
Distribution of the position of phenotypic and MMP alleles in the exons and protein domains of DAF-2. The *C. elegans daf-2* exons (introns have been excluded) are shown at left in black and gray, with the corresponding protein domains in color on the right. Schematic style adapted from De Meyts and Whittaker (2002). Mutations from previous phenotypic screens can be seen, with missense mutations in red and nonsense mutations in black; MMP alleles can be seen in blue on the right. Asterisks indicate which of the MMP alleles were found to have novel dauer phenotypes in this study. While the exons and protein domains are shown to scale, the positions of the lines indicating individual mutations are approximate.

**Figure 4:**
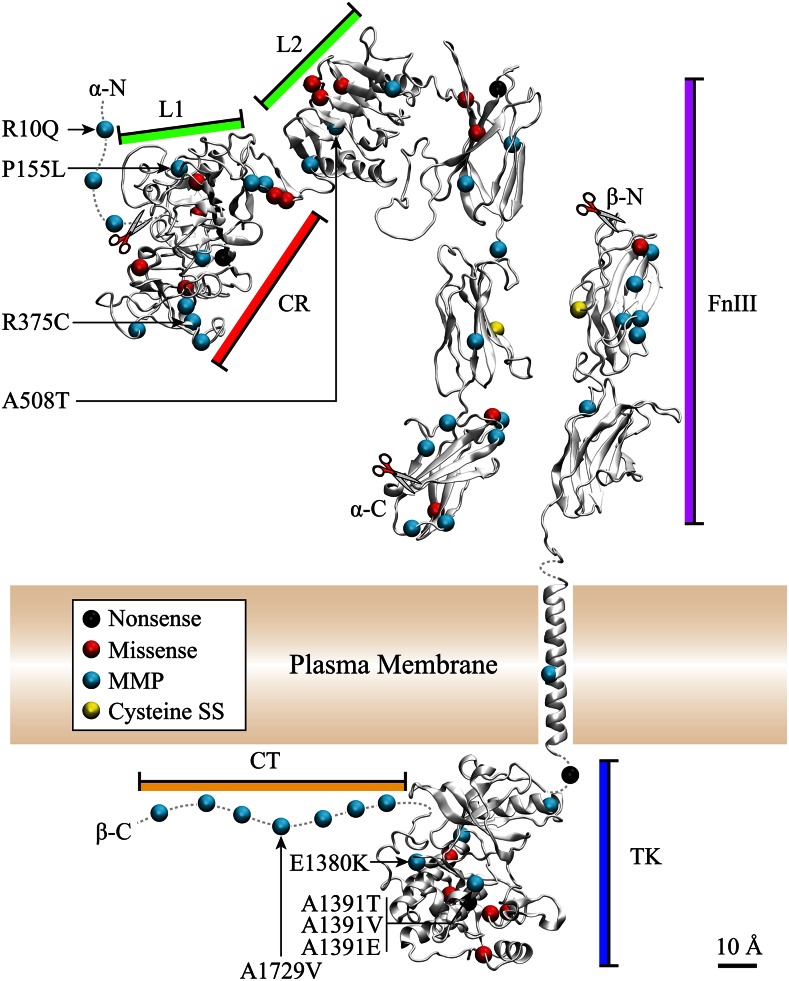
Novel DAF-2 structural model highlighting known mutations including newly characterized MMP alleles and their phenotypic consequences. A structural model of the DAF-2 insulin-like receptor is shown as a monomer based on domain-by-domain modeling using the online I-TASSER server (http://zhanglab.ccmb.med.umich.edu/I-TASSER/) and I-TASSER 2.1 (File S7, File S8, File S9, File S10, File S11, File S12, File S13, and Table S6) (Zhang 2008). Missense and nonsense mutations from phenotypic screens and MMP mutations are shown as indicated in the legend. The domain labels have been color-coded to correspond to the protein domains in Figure 3. The predicted signal peptide sequence is represented by an *N*-terminal dotted line with the cleavage site indicated at residue 143 (scissors). The position and number of Fibronectin Type III-like domains is uncertain due to an additional 75 amino acids that are present in DAF-2 when compared to human INSR (Figure 5). The putative furin-like cleavage site is at residue 930 (scissors). The receptor alpha and beta strands resulting from furin cleavage are each labeled at their N- and C-terminal ends (N-*α*, C-*α*, N-*β*, and C-*β*). The position and residue changes of MMP alleles resulting in a dauer phenotype are highlighted with black arrows and text. Only MMP mutations with a dauer phenotype and the three CRISPR-Cas9 mutations at A1391 are identified. Residue numbering refers to the preproreceptor. This method of displaying mutations was adapted from (Patel *et al.* 2008); the structure shown is a novel model of DAF-2 based on I-TASSER structural modeling.

The available *daf-2* MMP nsSNP alleles were distributed across the entire gene locus, with hits affecting exons encoding all major domains of the protein (Figure 3 and Figure 4). There was an average of 8.7 MMP mutations per kilobase in *daf-2* with hit frequencies similar in exons (10/kb) and introns (8.4/kb); a similar distribution was observed across other IIS pathway genes (Thompson *et al.* 2013). Complete loss of *daf-2* function results in embryonic lethality or early larval arrest (Gems *et al.* 1998); thus, none of the MMP alleles would be expected to be null on their own. It is possible that homozygous *daf-2* lethal alleles could be among the MMP collection due to coselection for second site suppressors of the lethality. Previous work has shown mutations in DAF-18/PTEN can act as such a suppressor (Ogg and Ruvkun 1998). In fact, three *daf-2* MMP strains we selected (VC40071, VC41001, and VC40691) also had nsSNPs in *daf-18*, which might allow the strains to survive the MMP room-temperature viability requirement, even with a loss-of-function *daf-2* mutation (Table S3). This highlights the value of the complete genome sequence, and the need to genetically outcross these strains prior to further study (see below, and *Materials and Methods*).

To determine the distribution of nsSNP alleles among evolutionarily conserved and nonconserved domains of DAF-2, we aligned related protein sequences both from within and between genera (File S1 and Figure S1). Six *Caenorhabditid* species with sequenced genomes were included in this analysis: *C. elegans*, *C. remanei*, *C. briggsae*, *C. brenneri*, *C. sp5*, and *C. tropicalis*. We also included genera that had well-annotated genomic, transcriptomic, and proteomic evidence for an insulin-like receptor (human, chimp, mouse, rat, frog, zebrafish, and fly). In order to correct for variable-sized insertions and deletions of nonconserved amino acids in DAF-2 relative to related receptors, T-COFFEE multiple-sequence alignments were performed with anchors at known functional sequence motifs surrounding clearly identifiable domains, including the insulin binding domain, fibronectin type III repeats, *trans*-membrane domain, and kinase domain (Morgenstern *et al.* 2006; Taly *et al.* 2011). This approach gave improved sequence alignments that allowed us to determine the degree of amino acid conservation both between and within genera (Figure S1, File S1, and Table 1).

**Table 1:** Conservation and phenotypic consequences of novel DAF-2 mutations

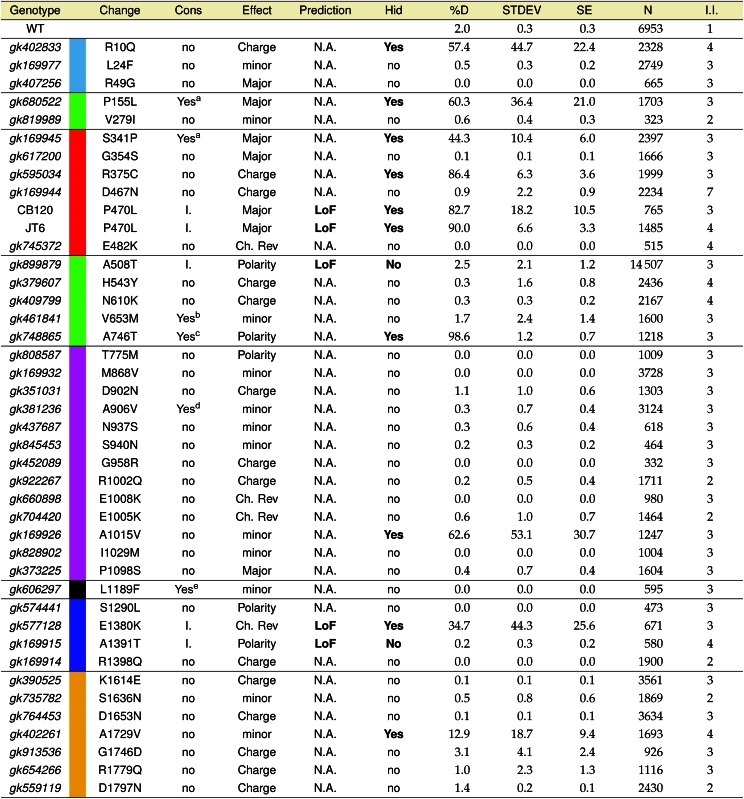

For each allele, the genotype, residue change (Change), degree of conservation (Cons), amino acid property change (Effect), and predicted phenotypic effect (Prediction) are indicated. In addition, high-temperature induction of dauer (Hid) phenotype, percent dauer (%D), standard deviation (STDEV), standard error (SE), number of worms assayed (N), and number of independent isolates (I.I.) are shown. Residue numbering refers to the preproreceptor.

Colors are shown as defined in Figure 3. WT, Wild Type; I., identical; Ch. Rev., charge reversal.

a Substituting residue is found at the corresponding location in a human insulin-like receptor.

b Substituting residue maintains conserved amino acid charge.

c Identical residue found in 22/27 aligned sequences (File S1).

d Conserved charge change.

e Substituting residue maintains hydrophobicity of the transmembrane domain.

As expected, the majority of MMP nsSNPs (28/40) were located in regions that were not evolutionarily conserved, in contrast to previous phenotypic screens that isolated mutations only in highly conserved domains of the DAF-2 receptor (Figure 2B). Among those SNPs in highly conserved domains, we found four altered residues that were identical when comparing human and *C. elegans* insulin receptors. These four allowed us to apply multiple algorithms, including PolyPhen2, SIFT, Align GVGD, and PROVEAN (Table S5), to predict the potential functional consequences of these substitutions (Kumar *et al.* 2009; Adzhubei *et al.* 2010; Hicks *et al.* 2011; Choi *et al.* 2012). Each of these four mutants, two within the insulin binding domain [(DAF-2(P470L), DAF-2(A508T)) and two within the tyrosine kinase domain (DAF-2(E1380K), DAF-2(A1391T)], was predicted by multiple analytic programs to be deleterious, suggesting they should result in a phenotype. For the remaining eight MMP alleles, which altered conserved nonidentical residues, the anchored sequence alignment and functional motif requirements of each domain were assessed. Three of these mutants [DAF-2(V653M), DAF-2(A906V), and DAF-2(L1189F)] were predicted not to affect function, because the missense mutation retained conserved hydrophobicity at the residue position (Table 1 and Table 2; asterisks). Similarly, three mutants [DAF-2(P155L), DAF-2(S341P), and DAF-2(A746T)] resulted in moderately conservative substitutions that might not be expected to affect function (Table 1; daggers). We concluded that the MMP *daf-2* allele collection provided a useful mix of amino acid substitutions, including several changes in conserved residues that had a high likelihood of affecting function and, therefore, would likely have a dauer-related phenotype.

**Table 2:** Validation of MMP phenotypes and modeling of conserved human disease alleles by CRISPR-Cas9

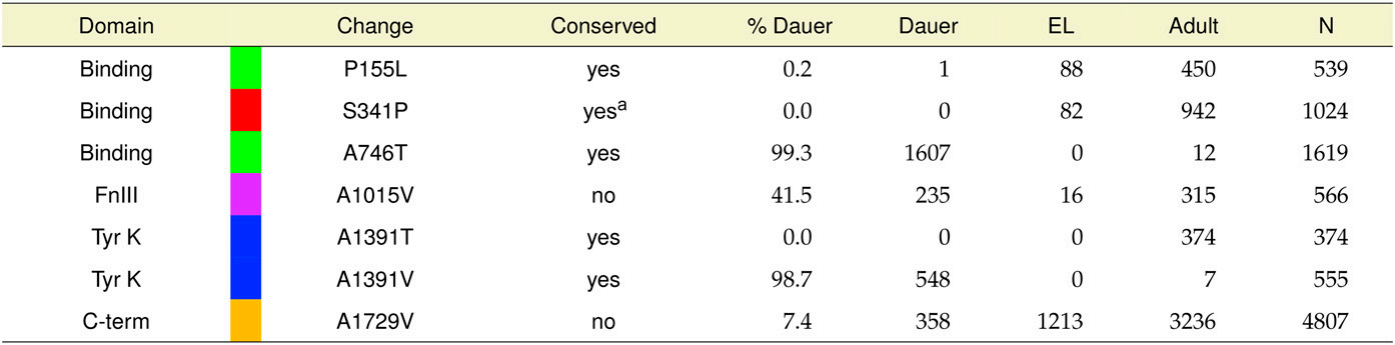

The domains, residue changes, conservation, dauer phenotype, and associated statistics for each of the engineered alleles are given. Residue numbering refers to the preproreceptor. Vertical colored bars reflecting different protein domains are consistent throughout tables and figures as defined in Figure 3. Dauer, number of dauer larvae; EL, number of embryonic lethal; Binding, ligand binding; FnIII, fibronectin Type III; Tyr K, tyrosine kinase; C-term, C-terminal.

a Substituting residue is found at the corresponding location in a human insulin-like receptor.

### Multiple MMP daf-2 alleles have a high temperature induction of dauer (Hid) phenotype

The large burden of mutations in the original MMP strains, visible in the genomic sequence data, often resulted in poor growth and high lethality; the number of nsSNPs per strain for the 40 *daf-2* allele-containing strains studied ranged from 198 to 1025 (mean ± SD of 471.6 ± 187). Two genetic backcrosses were performed for each MMP strain using a genetic marker for the *daf-2* region {*let-805*st456/*qC1* [*dpy-19(e1259) glp-1(q339)*]}, and at least two independent outcrossed isolates were selected for each allele. The independent outcrossed isolates allowed us to control for the influence of residual background mutations in our assays, a potential confounding factor given some strains had up to four additional mutations in genes known to affect dauer formation (Table S3). With a few exceptions (Table S2), none of the independent isolates after backcrossing had noticeable phenotypes at room temperature.

We screened each of the outcrossed MMP allele isolates for DAF-2 function by scoring for a high temperature induction of dauer (Hid) phenotype. High-temperature (27.2°) has previously been shown to induce dauers at low frequency in wild-type animals, and to enhance the dauer phenotype of weak DAF-2 loss-of-function alleles (Ailion and Thomas 2003). We also assayed two additional strains (CB120 and JT6) that were previously identified in genetic screens for constitutive dauers (Daf-c), but for which the molecular lesion was unknown; sequencing the *daf-2* locus in each strain allowed us to identify the causative mutation (see below).

Of the 42 strains harboring nsSNPs assayed for a Hid phenotype at 27.2°, four strains had a clear dauer phenotype (>80% dauer with SE <15), six strains had a variable dauer phenotype, and 32 strains had no observable dauer phenotype (<3.5% dauer with SE <2.5) (Table 1). All four strains resulting in a clear dauer phenotype had mutations in the insulin binding domain (amino acid residues 140–774). Two of these were novel MMP strains [DAF-2(R375C) and DAF-2(A746T)], and two were previously identified strains (CB120 and JT6), in which we identified the same DAF-2(P470L) mutation. Two of these three Hid-associated substitutions affected highly conserved amino acid residues. Alanine^746^ was conserved between 22 of the 27 receptors we aligned, with only 5/11 *Drosophila* species having a serine instead of an alanine. Proline^470^, altered in strains CB120 and JT6, was conserved in 25 of the 27 aligned species, with only the two *Branchiostoma* species having a glutamate at that position. The less-conserved residue change that also resulted in a clear dauer phenotype, DAF-2(R375C), was only conserved in four out of the six aligned *Caenorhabditids* (*C. elegans*, *C. remanei*, *C. briggsae*, and *C. sp5*), and 8/21 of the remaining aligned species, with 10 of the other species having either a lysine or a proline at this position. Thus, while strong evolutionary conservation of a residue was a very good predictor for mutations resulting in mutant phenotypes, substitutions in residues with weaker conservation also had functional consequences.

The six strains with variable dauer phenotypes affected amino acid residues dispersed across five different domains of the protein: DAF-2(R10Q) in the signal peptide, DAF-2(P155L) and DAF-2(S341P) in the insulin binding domain, DAF-2(A1015V) in the fibronectin type III domain, DAF-2(E1380K) in the tyrosine kinase domain, and DAF-2(A1729V) in the C-terminal domain. The signal peptide mutant, DAF-2(R10Q), was in a residue located after the first SL1 *trans*-splice site (Krause and Hirsh 1987), the major *trans*-spliced isoform, which is part of a potential (but experimentally unconfirmed) RXRR furin cleavage site. The remaining five variable Hid-associated strains similarly affected residues with only moderate-to-low conservation, weakening our confidence in attributing the phenotype to the *daf-2* nsSNP. The proline^155^ residue is conserved only among worms (*Caenorhabditids* and *Brugia*) with nonpolar amino acids (L, I, V, A) in the other aligned higher eukaryotes. Because leucine is found at this position in higher eukaryotes, the MMP DAF-2(P155L) mutation was not expected to yield a dauer phenotype. The other insulin binding domain substitution [DAF-2(S341P)] affected a serine conserved only in the two aligned *Xenopus* species, with higher eukaryotes (mouse, rat, chimp, and man) having a threonine at that position. The DAF-2(A1015V) substitution is in a residue that is neither conserved among worms nor the other aligned organisms. The DAF-2(E1380K) substitution affects a tyrosine kinase glutamate residue that is conserved only among *Caenorhabditids*, at the same site as the glutamine thought to anchor the E-helix to the F-helix in the human INSR. Finally, the DAF-2(A1729V) change had a weak dauer phenotype with a standard deviation greater than the mean percentage of dauers (12.9 ± 18.7%). This residue is only conserved among *Caenorhabditids*, and this region of the C-terminal domain is unique to this genus. Based on relatively weak sequence conservation of affected residues, we concluded that, with these results alone, we could not confirm that any of these substitutions cause the weak and variable dauer phenotypes.

The 32 MMP alleles that were indistinguishable from wild-type controls in our Hid phenotype assay were mostly in nonconserved residues distributed across the protein with two clear exceptions: DAF-2(A508T) in the insulin binding domain, and DAF-2(A1391T) in the tyrosine kinase domain. DAF-2(A1391T) was especially surprising since it was in a conserved catalytic loop amino acid residue found to be mutated in several patients with severe insulin resistance (Cama *et al.* 1993; Moreira *et al.* 2010). This mutation will be discussed below in the context of our results from CRISPR-Cas9 genome edited strains.

### Validating dauer phenotypes by CRISPR-Cas9 genome editing

To validate dauer phenotypes observed in the outcrossed strains, we carried out genome edits to mimic *daf-2* MMP alleles in an otherwise isogenic, wild-type genetic background using a *dpy-10* coediting strategy (Arribere *et al.* 2014). Genome editing using the CRISPR-Cas9 system has become robust for many, but not all, target sites in the *C. elegans* genome (Paix *et al.* 2014). We initially selected DAF-2 alterations R10Q, P155L, S341P, A746T, A1015V, E1380K, and A1729V for validation; we were successful in generating at least two independent strains with precise edits for all of these except R10Q and E1380K (Table S4).

Strains harboring the successfully CRISPR-Cas9 edited alleles were each assayed for a Hid phenotype, and the results compared to our genetic alleles (Table 2). As expected, the edited allele generating the insulin binding domain alteration A746T had a strong dauer phenotype, matching the MMP genetic results for this substitution. Two genomic edits resulting in substitutions (A1015V and A1729V) had moderate Hid phenotypes, also consistent with the MMP genetic alleles. Interestingly, the A1729V substitution had a consistently low percentage of dauer formation in both the MMP outcrossed strains (12.9%), and CRISPR-Cas9 edited strains (7.4%); we concluded this amino acid substitution was causative. This is the first dauer-enhancing allele identified in the DAF-2 C-terminal domain about which little is known functionally. Finally, two DAF-2 substitutions (P155L and S341P) had no Hid phenotype, in contrast to our genetic results. Thus, analysis of CRISPR-Cas9 edited alleles was essential in validating which of the MMP substitutions were causative for the dauer phenotype. Based on this analysis, seven of the assayed strains (five MMP, CB120, JT6) were validated as true Hid positives, while 35 MMP alleles resulted in a phenotype indistinguishable from wild type in the high temperature-induced dauer assay.

### C. elegans DAF-2 mutations faithfully model human disease alleles

Of the human INSR mutations associated with disease, five correspond to conserved residues in DAF-2 for which mutations have previously been identified that affect dauer formation (Table 3). A single MMP mutation [DAF-2(A1391T)] we assayed matched a known human INSR allele, but, surprisingly, resulted in no observable Hid phenotype. This DAF-2 variant aligned with both the INSR(A1135E) mutation found in a patient with Type A Insulin Resistance, and the compound heterozygous INSR(A1135V; N878S) mutation found in a patient with Rabson-Mendenhall Syndrome, a very severe form of insulin resistance. The lack of a Hid phenotype in this strain suggested that the *C. elegans* threonine substitution was tolerated despite the bioinformatic predictions to the contrary. This residue was chosen for further study to determine if mimicking the human disease alleles in *C. elegans* would provide additional insight.

**Table 3:** Phenotypic consequences of human INSR and corresponding *C. elegans* DAF-2 mutations

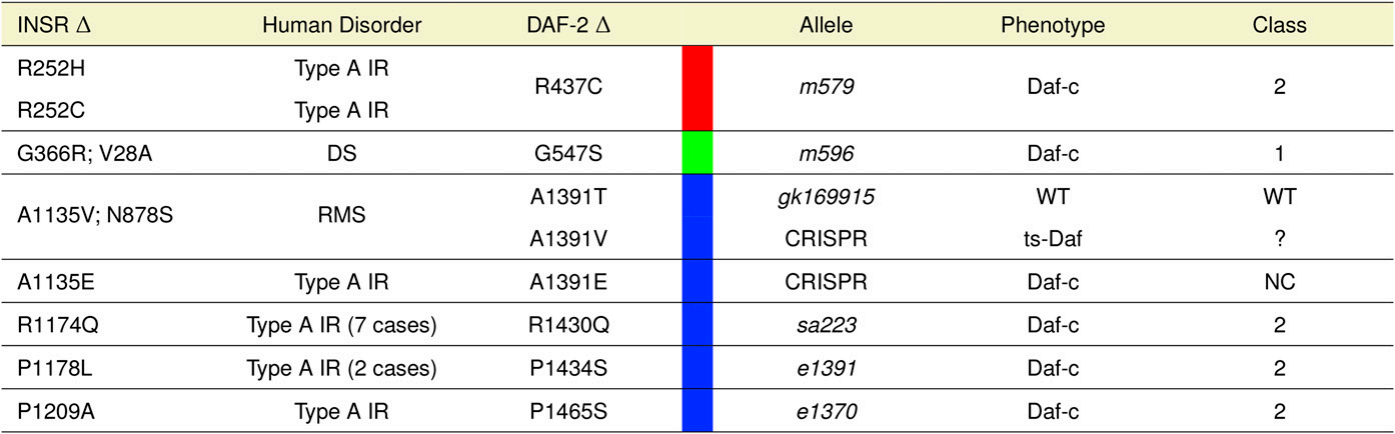

The human INSR residue change (INSR Δ), associated human disorder, worm DAF-2 residue change (DAF-2 Δ), worm allele name (Allele), associated worm phenotype, and class as defined in (Gems *et al.* 1998; Patel *et al.* 2008) are shown. Residue numbering refers to the preproreceptor. NC, nonconditional Daf-c; WT, wild-type; ?, unknown; DS, Donohue Syndrome; RMS, Rabson-Mendenhall Syndrome. Colors as described in Figure 3.

We generated three alanine^1391^ substitutions in *C. elegans* DAF-2 using CRISPR-Cas9 genomic editing, and assayed each for a Hid phenotype. One change [DAF-2(A1391T)] replicated the MMP allele that had no Hid phenotype. The other two changes [DAF-2(A1391V) and (A1391E)] mimicked human INSR substitutions resulting in disease. The DAF-2(A1391T) edited strains had no observable dauer phenotype, reproducing the results from the corresponding MMP strain, confirming that this substitution is tolerated in DAF-2. In contrast, the two other human disease substitution mimics had Hid phenotypes, although both the severity and penetrance of the phenotype differed between them. The DAF-2(A1391V) mutation resulted in 98.7% dauers at 27.2° and 0% dauer at 20°. Interestingly, the DAF-2(A1391E) edit could only be maintained as a heterozygote; animals harboring this edit gave rise to 25% dauer progeny at 15, 20, and 25°, suggesting the homozygous mutation had a Daf-c phenotype (Table 4). We confirmed this by genotyping individual progeny of these putative heterozygous animals, and found that 10 out of 10 sequenced individual dauer progeny were homozygous for the mutation and none of 30 sequenced nondauer progeny were homozygous for the mutation (only wild type, or heterozygous). Thus, the DAF-2(A1391E) homozygotes were found to be constitutive dauer at all temperatures tested, mimicking the severe phenotype observed in humans heterozygous for this same substitution. Importantly, our DAF-2 edits generated an allelic series for variants at this residue that were consistent with human disease severity: A1135T (tolerated), A1135V (moderate defect), and A1135E (severe defect).

**Table 4:** Genomic edits confirming an MMP allele and modeling a human allelic series at the conserved residue INSR(A1135)

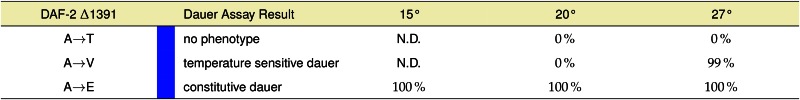

CRISPR-Cas9 was used to alter DAF-2(A1391) to the indicated residues and the results are shown for dauer assays performed at indicated temperatures. As observed with human INSR(A1135) mutations, these alteration result in a graded range of phenotypes, from no observable phenotype to constitutive dauer formation at all tested temperatures. N.D., not determined. Numbering refers to the preproreceptor. Color as described in Figure 3.

We explored these *daf-2* alanine^1391^ substitutions in more detail to understand the molecular basis of the lesions. Alanine^1391^ is located at the center of the tyrosine kinase active site, and is highly conserved. Sequence alignment between the HRD and DFG motifs, which define a key 23 amino acid long region of the tyrosine kinase active site (Wheeler and Yarden 2015), demonstrated that an alanine is found in 79 out of the 90 human tyrosine kinases we aligned; nine of 90 human tyrosine kinases have a threonine at this site, and only one (FAK2) has a valine (a possible loss-of-function variant) (File S6). Thus, like our *in vivo* results in *C. elegans*, sequence alignments suggest threonine may be a tolerated substitution at this position in humans.

To further understand the molecular consequences of substitutions at human INSR alanine^1135^ (corresponding to DAF-2 alanine^1391^), we turned to available structural data. Due to the numerous human diseases caused by tyrosine kinase dysfunction, several human tyrosine kinase crystal structures were available to explore the potential consequences of substitutions at this site (Berman *et al.* 2002). In the crystal structures for seven of the nine human tyrosine kinases with a threonine at this position, the threonine is stabilized through a hydrogen bond with a nearby glutamate (Table S7). To see if this might also be the case for the human INSR(A1135T) variant, CHARMM molecular dynamics simulation with periodic water solvation was performed for 20 ns on the human INSR tyrosine kinase crystal structure with the wild-type alanine, or an identical sequence with that residue substituted with a threonine (videos in File S5). We found that the wild type and A1135T variant INSR simulations resulted in no significant changes in the stability of the tyrosine kinase domain and active site configuration. We extended this analysis to determine the potential effects of the valine and glutamate substitutions at this residue that are associated with human disease. Although the valine change had no observable effects during simulation, the A1135E mutation significantly disrupted the active site stability. The human INSR(A1135E) has previously been shown to have processing defects in the endoplasmic reticulum (ER), preventing >95% of the receptor from reaching the cell surface (Cama *et al.* 1993). Our simulation demonstrated that any INSR(A1135E) making it to the cell surface would likely be compromised for function, based on a very unstable kinase active site.

## Discussion

### An emerging spectrum of human insulin receptoropathies

Over 150 different alleles have already been described in the human insulin receptor; this list is likely to expand greatly in the near future owing to the ever-decreasing cost of acquiring genome sequence information, and the many efforts world-wide in collecting large cohorts of sequenced genomes. A key challenge for the medical research community is to identify those alleles that are associated with disease risk, and tailoring individual therapy for the best patient outcome.

Like many conserved pathways, dysfunction of IIS can result in a range of disease phenotypes depending on the nature of the lesion. Thus, the phenotypic consequences of INSR variants are graded from none to very severe, reflecting the extent to which signaling is compromised. It is generally accepted that INSR polymorphisms retaining >50% receptor function do not result in human disease (Kadowaki *et al.* 1988; Taylor *et al.* 1992; Semple *et al.* 2011, 2016; Semple 2016). However, available studies suggest that reduction of INSR function to the ∼25–50% range results in Type A Insulin Resistance (TA-IR), in which patients present with hyperinsulinemia, acanthosis nigricans, polycystic ovarian syndrome (PCOS), hirsuitism, and hyperandrogenism. Patients with <25% INSR function have the more severe disorders Rabson Mendenhall syndrome (RMS) and Donohue syndrome (DS; formerly “leprechaunism”). Patients with RMS and DS have additional symptoms of linear growth retardation, impaired development of skeletal muscle and adipose tissue, hypertrichosis, and additional dysmorphic features. While patients with TA-IR have normal lifespans, RMS and DS patients have a much worse prognosis, typically living 3–17 yr for RMS, and <3 yr for DS. These clinical outcomes underscore the importance of fully understanding the functional consequences of INSR mutations.

### C. elegans as a model for insulin-like signaling

The worm has proven to be a genetically amenable model for examining conserved biological pathways, such as the insulin-signaling pathway, which is deregulated in many human diseases. Worm genetics provided an early framework for understanding the order of this complex signaling cascade. We developed the pipeline shown in Figure 1 to determine if *C. elegans* genetic tools could (1) improve our understanding of the well-studied insulin-like receptor DAF-2, and (2) prove useful for the analysis of human metabolic disease for which sequence polymorphisms were readily available from exon-sequencing efforts. Using a positive selection screen for *daf-2* mutants, David Gems’ group has investigated the molecular basis of the different alleles resulting in a dauer-associated phenotype. Their analysis resulted in the clustering of existent genetic alleles into two classes (I and II). Furthermore, they developed an online tool (RILM) that contains a sequence alignment across many species, allowing them to note that the two Class I mutants *daf-2*(*m577*) and *daf-2*(*m596*) occur at residues with known human insulin receptor disease-causing mutations, both of which are thought to cause processing defects. Here, we show numerous other Class II *daf-2* mutants that also occur in known human disease-causing residues (Table 3). While class distinction has proven useful for the more severe Daf-c causing mutations, this distinction has not been made for the weaker high-temperature induction of dauer (Hid) mutants presented in this study. Our results demonstrate that some human disease-associated allele mimics in DAF-2 present with weaker phenotypes, highlighting the importance of the biological assay and its sensitivity in interpreting function.

Our study also tested the utility of the MMP resource in providing new insights into the highly conserved and well-studied IIS pathway. Phenotyping 40 *daf-2* MMP alleles using a relatively simple and quick high temperature-induced dauer assay, we identify five new dauer alleles, and 35 tolerated DAF-2 substitutions (Table 1). We found that accurately predicting phenotypes for animals harboring even severe amino acid substitutions (*e.g.*, charge reversal) was not possible, with many nonconservative changes resulting in no phenotype, whereas even conservative changes could lead to dauer phenotypes. Our study expands the regions of DAF-2 explored by mutagenesis for functional consequences, while revealing clear parallels to human disease alleles in the evolutionarily conserved IIS pathway.

### Human disease alleles in the conserved INSR receptor are effectively modeled in C. elegans

One of the twice-backcrossed MMP *daf-2* alleles lacking a Hid phenotype [*gk169915*, DAF-2(A1391T)] aligns with known human disease alleles at the corresponding position in INSR (A1135V in a complex heterozygote; A1135E in a heterozygote) (Cama *et al.* 1993; Moreira *et al.* 2010). The absence of a Hid phenotype in *C. elegans* for DAF-2(A1391T), which we confirmed by genomic editing in an otherwise wild-type background, is surprising because of the change in charge for this conserved residue within the tyrosine kinase domain that is predicted to be more deleterious than the A to V change previously associated with human disease. Our genetic results, coupled with the observation that the alanine to threonine change is present in 10% of the 90 human tyrosine kinase domains we inspected (File S6), suggests it may similarly be a tolerated change in the human INSR (Manning *et al.* 2002). Further analysis of this alanine^1391^ residue demonstrated our ability to leverage the relatively quick genomic editing in *C. elegans* to model multiple alleles associated with human disease at the corresponding position in INSR. In fact, the edited substitutions in *C. elegans* generate an allelic series of phenotypic severity, matching, and further clarifying, that observed in human patients.

The human A to V^1135^ INSR mutation has only been observed as one of the components of a compound heterozygous mutation: INSR(N851S; A1135V). As RMS is seen only with either homozygous INSR mutations or compound heterozygous mutations, it can be assumed that both mutations cause a significant loss of function. However, the degree of loss of function due to each allele is still unknown. The A to E substitution at the same residue has already been shown in cell culture to cause misfolding of the protein in the ER, resulting in only a small fraction of this protein reaching the plasma membrane to interact with ATP (Cama *et al.* 1993). Whether the A to V substitution causes a similar effect is unknown. Notably, the A to T^1135^ INSR mutation has not yet been observed in humans, even though 10% of human tyrosine kinases have a threonine at this position. Our results in *C. elegans* have demonstrated that these three substitutions constitute an allelic series, strongly suggesting that human disease severity results from an analogous graded effect of these changes on INSR function.

The available crystal structures of human tyrosine kinases and molecular dynamics simulations provide an approach to understanding how mutations may induce graded effects on tyrosine kinase function (Table S7). By replacing the A^1135^ INSR residue with a threonine, the ∼3.0 Å distance between the substituted threonine’s hydroxyl group and the carboxylic acid group of E^1201^ suggested a potential hydrogen bond stabilizing the two charged residues in a relatively otherwise hydrophobic pocket of the protein. In the 10% of human tyrosine kinases normally containing a threonine corresponding to the A1135 INSR residue, a hydrogen bond can be seen in seven crystal structures, providing further support for the INSR hydrogen bond explanation for how the threonine replacement could occur without significantly altering the stability of the protein. CHARMM molecular dynamics simulations of all three missense mutations in the human INSR confirmed the T^1135^–E^1201^ hydrogen bond and demonstrated that the A to T and A to V changes retained active site stability, while the A to E change did not (videos in File S2, File S3, File S4, and File S5). Thus, analysis and modeling of these three substitutions provides an explanation for the genetic results observed in *C. elegans*, and the degree of disease severity in humans.

Misfolding of the INSR in the ER has been shown in humans to mediate many of the loss-of-function mutations (Taylor 1992). It would be interesting to directly address DAF-2(A1391E) protein folding, trafficking, and membrane localization; however, suitable reagents for these type of experiments have yet to be developed in *C. elegans*. Augmenting the facile genetics of the worm system with *in vivo* DAF-2 cell biology will greatly advance our understanding the molecular mechanisms of dysfunction and add another dimension to modeling human INSR disease alleles.

### Residue conservation predicts phenotypic consequences for mutations

The MMP resource allows us to address the question of whether past phenotypic selections biased our collection of mutants affecting DAF-2 function. It did not; mutations in highly conserved residues are very likely to have phenotypic consequence. Five previously identified genetic alleles resulting in dauer formation defects in *C. elegans* result from substitutions in residues conserved in human INSR; our current study adds another two to the list (Table 3).

Changes to evolutionarily constrained residues in the insulin-like receptor DAF-2 have phenotypic consequences in *C. elegans* that correlate well with disease severity in humans with corresponding alterations. Of the five previously identified genetic reduction-of-function alleles in *daf-2*, each alters an amino acid residue that is identical to either 27/27 or 26/27 related receptors from multiple species that we aligned. The classic *daf-2*(*e1370*) allele encodes DAF-2(P1465S) that aligns with the INSR(P1209A) mutation in a patient with Type A Insulin Resistance (TA IR); this proline residue is on the *α*F-helix of the tyrosine kinase domain. The *daf-2*(*e1391*) allele encodes DAF-2(P1434S) that aligns with the INSR(P1178L) mutation located in the middle of the activation segment of the tyrosine kinase domain and is commonly mutated in patients with TA IR. The *daf-2*(*sa223*) allele encodes DAF-2(R1430Q) that perfectly matches the INSR(R1174Q) amino acid change found in seven patients with TA IR. The *daf-2*(*m579*) allele encodes DAF-2(R437C) that perfectly matches a patient with TA IR with an INSR(R252C) mutation and aligns with the INSR(R252H) mutation in another patient with TA IR. This arginine is in the insulin-binding domain immediately adjacent to a completely conserved cysteine that likely plays a role in folding and may participate in an intrachain disulfide bond. The *daf-2*(*m596*) allele encodes DAF-2(G547S) that aligns with a compound heterozygous patient with INSR(G366R; V28A) mutations resulting in DS—the most severe form of human insulin resistance. Of the aligned receptors, this glycine is conserved in the insulin binding domains of human, chimp, mouse, rat, frog, and zebrafish, and five out of six of the *Caenorhabditids* (*C. briggsae* had a glutamate at this residue). Thus, the most highly conserved domains of the INSR and DAF-2 are functionally dependent on the primary amino acid sequence, and substitutions have a high likelihood of resulting in dysfunction; a similar conclusion was reached by (Ng and Henikoff 2006).

Our current results using the MMP resource further support these conclusions, but also reveal at least one major surprise. Most of the DAF-2 substitutions we assayed for a Hid phenotype, a single yet sensitive metric for function, were wild type and in nonconserved residues. Therefore, the large majority of alleles following mutagenesis and selection only for viability have no obvious deleterious effect on DAF-2 function. As described above, one of these (DAF-2(A1391T)) was, nonetheless, very informative in suggesting a functionally tolerated change that is unlikely to result in human disease if identified in INSRs. The three MMP alleles that did result in a Hid phenotype identify novel residues in which alterations would be predicted to have functional consequences for the human INSR, and have a high likelihood of being associated with human disease; we would expect disease severity to correspond to the strength of the Hid phenotype in *C. elegans*.

### Novel genetic insight into the C-terminal domain of DAF-2

**Figure 5:**
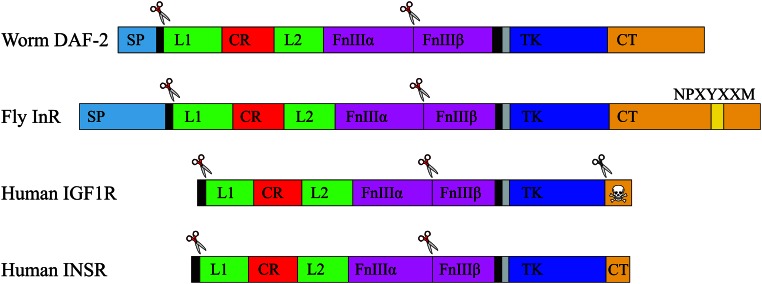
Conserved and divergent domains of four insulin-like preproreceptors. Protein schematics were drawn to scale relative to the number of amino acids in each domain. The worm DAF-2 domains were mapped onto the other receptors using anchored T-COFFEE alignment (File S1). The Fly InR C-terminus contains multiple NPXYXXM motifs, which can bind both IRS-like proteins and PI3K (Poltilove *et al.* 2000). The Human IGF1 “dependence receptor” has a proapoptotic C-terminus (skull and crossbones) (O’Connor *et al.* 1997; Hongo *et al.* 1998; Liu *et al.* 1998; Goldschneider and Mehlen 2010). Unless indicated otherwise, domain designations are as shown in Figure 3.

One of the dauer-enhancing MMP alleles, DAF-2(A1729V), confirmed by CRISPR-Cas9 genome editing, is located in the C-terminal domain, a stretch of 658 amino acids lacking previous genetic hits. Although present in all INSR-related receptors, the C-terminal domain of the protein displays little to no conservation in its amino acid sequence or its length (Figure 5). The human IGF-1 receptor has previously been shown to act as a dependence receptor, with the release of proapoptotic peptide cleavage products of the C-terminal domain during an absence of growth factor signaling (O’Connor *et al.* 1997; Hongo *et al.* 1998; Liu *et al.* 1998; Goldschneider and Mehlen 2010). In flies, the C-terminal domain of InR has the properties of Insulin Receptor Substrate proteins (IRSs) that, in humans, are encoded by four distinct genes (Poltilove *et al.* 2000). Fusion of the intracellular domains of the *Drosophila* InR protein to the human INSR ectodomain rescues the loss of INSR signaling in a mammalian cell line lacking all four human IRS proteins. Interestingly, knockout of the sole fly ortholog of IRSs, Chico, results in a completely different nonlethal phenotype than the InR knockout in flies. Therefore, while the C-terminal domain of InR in *Drosophila* can substitute functionally for absent human IRS proteins in tissue culture systems, there may be additional *in vivo* functions of this domain in flies that are unrelated to canonical IIS.

Results from *Drosophila* may be relevant to understanding a role for the *C. elegans* DAF-2 C-terminal domain. DAF-2 and fly InR share many characteristics, including being the sole insulin-like receptor, and both possess a long C-terminal domain with numerous tyrosine residues (Figure 5). Like flies, *C. elegans* has a single ortholog of IRSs, IST-1. Mutations in *ist-1* can enhance the dauer phenotype, but only when combined with *daf-2* loss-of-function mutations (Wolkow *et al.* 2002), suggesting that IST-1, like Chico in flies, is not essential for signaling through the IIS pathway. Thus, it seems plausible that the worm might share yet another feature with the fly: the ability to signal directly through the C-terminal domain instead of requiring recruitment of IRS proteins to the plasma membrane. Our characterization of a weak dauer-enhancing MMP allele that results in DAF-2(A1729V) demonstrates for the first time that the C-terminal domain is required for proper IIS signaling.

### Summary

In this study, insulin-like receptor signaling was interrogated by characterizing a rich array of novel *C. elegans* mutations, making comparisons to known human disease-causing insulin receptor alleles, and using whole animal *in vivo* functional assays of allele variants. Capitalizing on the strengths of the worm system, including forward genetics, existing defined gene alleles, and relatively easy genomic editing by CRISPR-Cas9, we were able to quickly phenotype 40 novel alleles, while also creating and assaying human allele mimics. Our results were a mix of the expected and unexpected, highlighting the importance of testing allele function in the context of a whole animal system in which receptor processing and function are integrated with the entire metabolic program. Our experiences with the insulin-like signaling pathway in *C. elegans* demonstrates the power of this model system to better understand the possible organismal-level consequence of mutations in conserved physiological processes. Facile CRISPR-Cas9 genomic editing makes feasible the assessment of any suitable human allele suspected of disrupting gene function in a model system for which a robust assay exists or can be developed. This opens new possibilities for interpreting the increasing volumes of human genome sequence data for identify mutations contributing to human diseases.

## Supplementary Material

Supplemental material is available online at www.g3journal.org/lookup/suppl/doi:10.1534/g3.116.037184/-/DC1.
