# Supplementary material for: Caenorhabditis elegans DAF-2 as a Model for Human Insulin Receptoropathies
Source: G3 (Bethesda). 2016 Nov 15;7(1):257–68. doi: 10.1534/g3.116.037184 (PMC5217114; doi:10.1534/g3.116.037184)
Supplement: Supplementary file 1 [file 257FigureS1.docx]

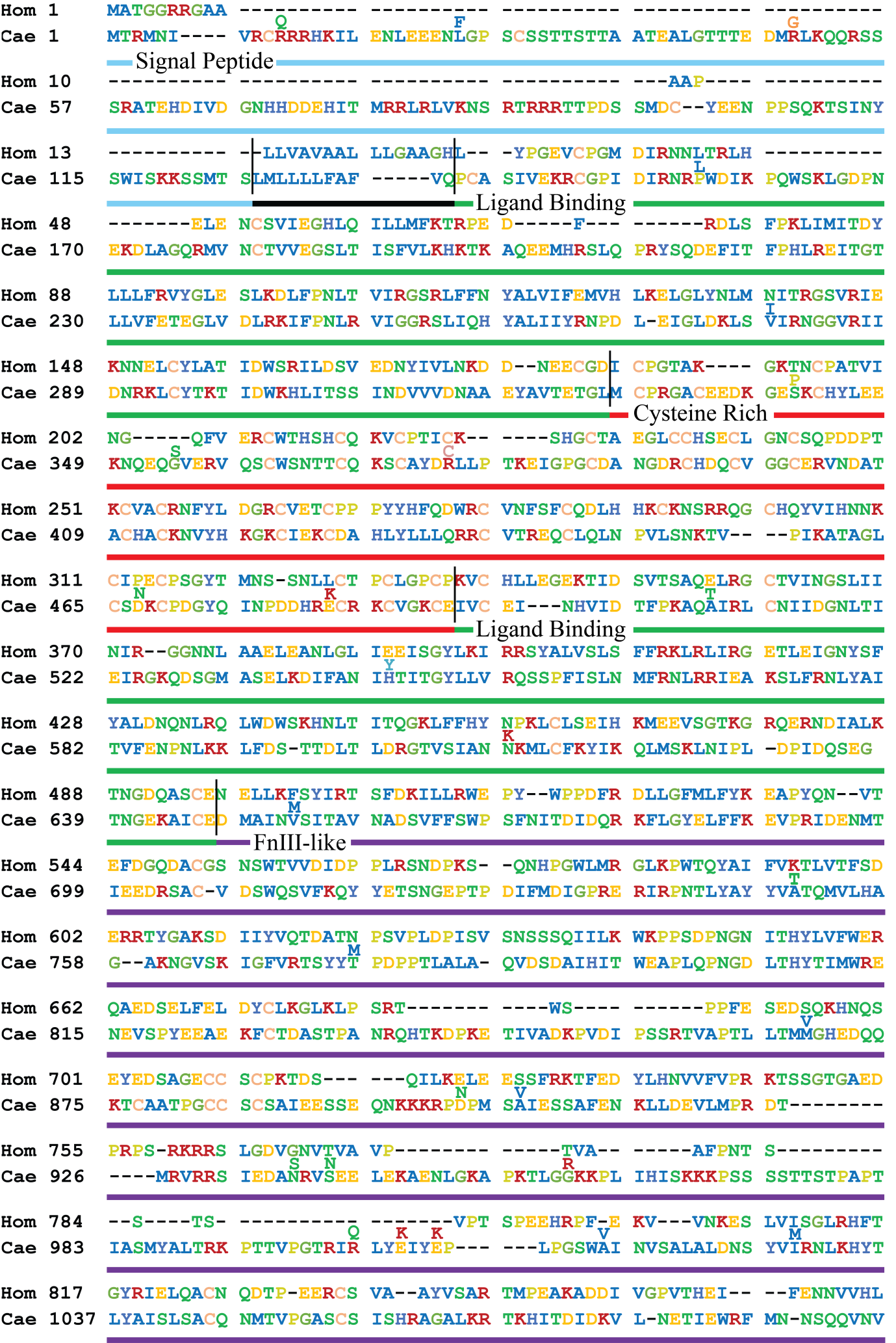


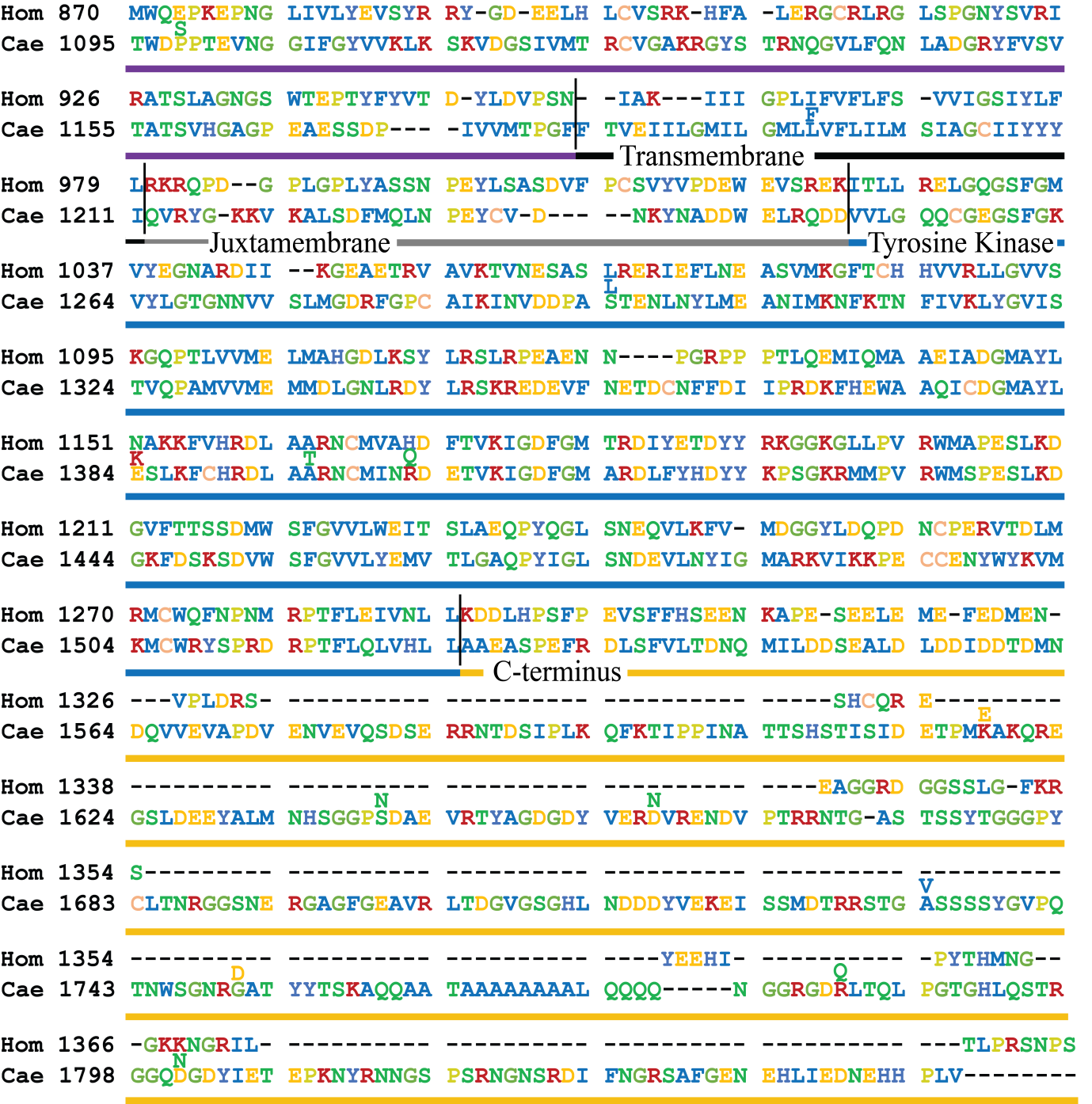


**Figure S1. Anchored T-COFFEE sequence alignment of human and *C. elegans* insulin/insulin-like receptors.** Amino acid number refers to the previous amino acid if the row starts with a gap and major domains indicated with a colored underlined as defined in the figure. Million Mutation Project (MMP) alleles are shown with the alternative amino acid in between the *C. elegans* and human reference amino acids. Underline colors described in Table 1. Amino acid colors as used in Seaview ([Gouy *et al.* 2010](#_ENREF_1)).

**Literature Cited**

Gouy, M., S. Guindon and O. Gascuel, 2010 SeaView Version 4: A Multiplatform Graphical User Interface for Sequence Alignment and Phylogenetic Tree Building. Molecular Biology and Evolution 27**:** 221-224.
