# Supplementary material for: Caenorhabditis elegans DAF-2 as a Model for Human Insulin Receptoropathies
Source: G3 (Bethesda). 2016 Nov 15;7(1):257–68. doi: 10.1534/g3.116.037184 (PMC5217114; doi:10.1534/g3.116.037184)
Supplement: Supplementary file 10 [file 257FileS5.docx]

**File S5** High resolution video of molecular dynamics simulation highlighting the ATP movements in the tyrosine kinase binding pocket of normal human INSR(A1135E). (.avi, 15.6 MB)

Available for download as a .avi file at <http://www.g3journal.org/lookup/suppl/doi:10.1534/g3.116.037184/-/DC1/FileS5.avi>
