# Supplementary material for: Caenorhabditis elegans DAF-2 as a Model for Human Insulin Receptoropathies
Source: G3 (Bethesda). 2016 Nov 15;7(1):257–68. doi: 10.1534/g3.116.037184 (PMC5217114; doi:10.1534/g3.116.037184)
Supplement: Supplementary file 22 [file 257FileS11.docx]

**File S11** DAF-2 I-TASSER model PDB file: ectodomain part 4. (.zip, 47.2 KB)

Available for download as a .zip file at http://www.g3journal.org/lookup/suppl/doi:10.1534/g3.116.037184/-/DC1/FileS11.zip
