# Supplementary material for: Caenorhabditis elegans DAF-2 as a Model for Human Insulin Receptoropathies
Source: G3 (Bethesda). 2016 Nov 15;7(1):257–68. doi: 10.1534/g3.116.037184 (PMC5217114; doi:10.1534/g3.116.037184)
Supplement: Supplementary file 28 [file 257TableS1.docx]

**Table S1** Primers used for *daf-2* sequencing. (.xlsx, 11.4 KB)

Available for download as a .xlsx file at http://www.g3journal.org/lookup/suppl/doi:10.1534/g3.116.037184/-/DC1/TableS1.xlsx
